# Maize Plant Resilience to N Stress and Post-silking N Capacity Changes over Time: A Review

**DOI:** 10.3389/fpls.2016.00053

**Published:** 2016-02-09

**Authors:** Sarah M. Mueller, Tony J. Vyn

**Affiliations:** Agronomy Department, Purdue UniversityWest Lafayette, IN, USA

**Keywords:** maize, genotype era, nitrogen stress tolerance, post-silking nitrogen, nitrogen nutrition index

## Abstract

We conducted a synthesis analysis on data from 86 published field experiments conducted from 1903 to 2014 to explore the specific consequences of post-silking N accumulation (PostN) in New Era vs. Old Era hybrids on grain yield (GY) and recovery from plant N stress at flowering (R1 stage). The Old Era encompassed studies using genotypes released before, and including, 1990 and the New Era included all studies using genotypes released from 1991 to 2014. Mean N fertilizer rates for experiments in the Old and New Era were similar (170 and 172 kg ha^−1^, respectively), but plant densities averaged 5.0 plants m^−2^ in the Old Era vs. 7.3 plants m^−2^ in the New Era studies. Whole-plant N stress at R1 for each hybrid, environment and management combination was ranked into one of three categories relative to the N Nutrition Index (NNI). The key findings from this analysis are: (i) New Era genotypes increased the proportion of the total plant N at maturity accumulated post-silking (%PostN) as N stress levels at R1 increased—demonstrating improved adaptability to low N environments, (ii) New Era hybrids maintained similar GY on a per plant basis under both low and high N stress at R1 despite being subject to much higher population stress, (iii) PostN is more strongly correlated to GY (both eras combined) when under severe R1 N stress than under less acute N stress at R1, (iv) the New Era accumulated more total N (an increase of 30 kg N ha^−1^) and higher %PostN (an increase from 30% in Old to 36% in New Era), and (v) the change in stover dry weight from silking to physiological maturity (ΔStover) has a positive, linear relationship with PostN in the Old Era but less so in the New Era. This increased understanding of how modern genotypes accumulate more N in the reproductive stage and have more PostN and GY resilience to mid-season N stress, even when grown at much higher plant densities, will assist trait selection and N management research directed to improving maize yields and N efficiencies simultaneously.

## Introduction

Physiological changes associated with increased maize grain yield over time have increased the duration of the effective growing season allowing the maize crop more time to accumulate photosynthates and, therefore, biomass. Earlier planting dates (Kucharik, 2006, 2008) and physiological changes such as increased duration of leaf photosynthesis, delayed leaf senescence, and increased biomass accumulation during the grain filling period (Ma and Dwyer, 1998; Echarte et al., 2008) have occurred simultaneously with enhanced tolerance to abiotic stresses such as drought (Byrne et al., 1995), crowding stress (Tollenaar and Lee, 2002), and low soil N (McCullough et al., 1994; Tollenaar and Wu, 1999). As discussed by Bender et al. (2013), the increased length of the growing season also increases the importance of season-long nutrient availability for accumulation and utilization by the plant. Because of the important role the N plays in enhancing grain yield (Anderson et al., 1985), the dynamics of crop N uptake and partitioning over the length of the season has been of great interest. Characterization of how the timing of plant N uptake has changed with current vs. older genotypes increases our understanding of how to further improve GY through N accumulation, as well as how to most efficiently apply N fertilizer.

While grain filling in maize relies almost entirely on concurrent photosynthesis for its carbohydrate requirements (Rajcan and Tollenaar, 1999a,b; Borrás et al., 2004; Tollenaar et al., 2004; Lee and Tollenaar, 2007), the required N for grain filling cannot be met by concurrent new N assimilation during reproductive growth and must be supplemented by remobilizing N assimilated in vegetative organs prior to silking (Swank et al., 1982; Ta and Weiland, 1992; Rajcan and Tollenaar, 1999a; Hirel et al., 2007). Grain N can arise from either this remobilized N (RemobN) or new N taken up during the grain filling period (PostN; Christensen et al., 1981; Crawford et al., 1982; Pan et al., 1986; Chapin et al., 1990; Cliquet et al., 1990; Ta and Weiland, 1992; Moll et al., 1994; Chen et al., 2015b). The proportions each of these sources contribute to the GrainN appears to be antagonistic (Pan et al., 1984, 1995; Coque and Gallais, 2007; Ciampitti and Vyn, 2013). There has been much interest and speculation on the role of PostN in maize, but previous research has shown mixed conclusions as to its impact on final GY. However, there is consensus on the importance of the relationship between PostN and the stay-green trait. Stay-green, or increased leaf longevity, is often associated with greater PostN accumulation which allows for delayed remobilization of N from the leaves (Rajcan and Tollenaar, 1999b; Mi et al., 2003; He et al., 2004; Pommel et al., 2006; Ciampitti and Vyn, 2011; Ciampitti et al., 2013). Stay-green is often cited as a major factor for increased GY in modern hybrids (Fakorede and Mock, 1980; Duvick, 1984; Ma and Dwyer, 1998; Pommel et al., 2006), and is dependent on the balance between PostN and RemobN (Wang et al., 2014; Chen et al., 2015b). Up to 70% of N in the leaves is associated with the chloroplasts (Gan and Amasino, 1997) and for this reason, leaf N status, leaf longevity, and photosynthetic activity are interrelated (Novoa and Loomis, 1981; Mi et al., 2003). In addition to improved genetics, stay-green may also be the result of improved management-such as with the use of foliar fungicides-if they improve leaf health and delay leaf senescence.

The drivers of increased PostN appear to be regulated by both the genotype (Below et al., 1981; Moll et al., 1982; Osaki, 1995; Oikeh et al., 2003; Uribelarrea et al., 2007) and the environment (Swank et al., 1982; Mackay and Barber, 1986; Bundy and Carter, 1988). Several studies have found PostN to decline as grain sink-size increases because of the inherent competition between the roots and developing ear for photoassimilates (Pan et al., 1984; Anderson et al., 1985). In contrast, Uribelarrea et al. (2004) found that PostN increased with the increased sink demand of high protein maize. While some studies have found a positive relationship between GY and PostN (Karlen et al., 1987; Akintoye et al., 1999; Worku et al., 2007; Liu et al., 2014; Yan et al., 2014), other studies have found no relationship between PostN and GY (Beauchamp et al., 1976).

The need to improve nitrogen use efficiency (NUE) in maize cropping systems because of economic and environmental concerns is not a new topic, but is still a great challenge. NUE is comprised of both N recovery efficiency (NRE) and N internal efficiency (NIE). It has been reported that the relative importance of NRE compared to NIE in determining NUE may differ depending on N supply to the crop. Moll et al. (1982) and Ma and Dwyer (1998) reported that under low N supply variation among genotypes for NUE was largely due to differences in NIE, whereas under high N conditions, differences in NUE were mostly attributed to differences in NRE. In contrast, Kamprath et al. (1982) hypothesized that NRE would be more important under low N and NIE would be of greater advantage under high N conditions. The rational for the latter hypothesis is that when N supply is not limiting, even hybrids that are less efficient at accumulating N will still take up adequate N, and hybrid separation in NUE will instead come from the genotype's ability to use accumulated N for grain production. Even 30 years ago, however, it was recognized that improvement in both NRE and NIE are important for the further enhancement of NUE (Anderson et al., 1985). Conclusions about the role of NIE are very hybrid dependent; a recent review observed substantial improvement in average NIE (from 49.7 to 56.0 kg grain kg^−1^ above-ground plant N uptake) in maize experiments involving hybrids after 1990 vs. before 1990 (Ciampitti and Vyn, 2012).

Results are also mixed in terms of the role that PostN plays in increasing NUE and its components. PostN has been found to increase NUE (Coque and Gallais, 2007) and NRE (Worku et al., 2007; Wang et al., 2014), although Moll et al. (1982) found no influence of PostN on NUE in earlier maize genotypes. The indirect role of the stay-green trait in achieving improvements in NUE has been observed in modern hybrids (Peng et al., 2010; Wang et al., 2014).

One useful metric for determining in-season crop N status is the N Nutrition Index (NNI) developed by Lemaire et al. (1989). The NNI is based on the stable relationship between crop biomass and optimal N concentration and takes into consideration the ratio of actual N content compared to optimal N content for a given crop biomass (Lemaire et al., 1996). It has been determined that an NNI > 1 indicates crop biomass growth is not limited by N supply while an NNI < 1 is evidence of N deficiency (Sadras and Lemaire, 2014). In maize, several studies have found NNI to be a sensitive indicator of crop N status during vegetative and onset of reproductive growth stages (Plénet and Lemaire, 2000; Herrmann and Taube, 2004; Lemaire et al., 2008a; Ziadi et al., 2008). Ziadi et al. (2008) found NNI to be a better predictor of crop N status and final grain yield than either chlorophyll meter readings or N concentration of the uppermost collared leaf. Likewise, Ciampitti et al. (2012) also found NNI to be correlated with relative grain yield and plant N uptake at the silking stage.

We conducted a synthesis analysis in order to better understand the changing dynamics of maize N uptake over time, and how these changes in crop accumulation and utilization of N impacts GY, N efficiency, and maize plant response to N stress. Earlier synthesis analyses have recently explored the change over time in maize N use efficiencies and grain N sources (Ciampitti and Vyn, 2012, 2013). Our intention was to specifically study the change in PostN over time and how this has impacted maize response to mid-season N stress and plant N dynamics. Utilizing all of the known, published materials meeting our minimal inclusion criteria, we compiled a data set to answer the questions: (1) How have the changes in PostN accumulation by modern hybrids contributed to changes in grain yield per plant and per unit area?, (2) Does the level on N stress at R1 impact post-silking N and dry matter accumulation differently in modern vs. older hybrids?, and (3) Have the previously documented relationships between PostN accumulation and post-silking dry matter gains plus plant N content distribution at maturity changed with the improvement of maize hybrids?

## Materials and methods

### Data selection

Data for this synthesis analysis was obtained from peer-reviewed journals and publically available thesis dissertations (Supplementary Tables 1, 2). All values utilized were treatment means (further divided into means of year, location, genotype, etc.), regardless of replication number. The minimum criterion for inclusion was the reporting of above ground whole-plant N accumulation at both R1 (flowering) and R6 (physiological maturity) growth stages. Treatments that employed artificial interventions during the growing season to alter the source to sink ratio were not included. Data values were procured from tables and, if necessary, from digitized figures. For all measurements, only above ground plant tissue was considered in this review. In a few cases, multiple papers were published from the findings of a single field experiment. In these situations, only unique treatment data means were utilized in our synthesis analyses.

When necessary, values not explicitly stated, but that could be calculated based on reported equations for relationships between grain yield, plant N uptake, or plant biomass accumulation, were computed arithmetically. All data sets were converted to the same scale, g plant^−1^ (_P_) or kg ha^−1^ (_A_), with use of the reported plant population.

Genotypes included in this synthesis analysis were predominately single-ear hybrids, although there are some semi-prolific hybrids, inbred lines, and open pollinated cultivars also represented. Environments varied widely. Of the 86 unique experiments incorporated, 43% of experiments were conducted in the United States, 32% in China, and the remainder in other countries including France, Canada, Zimbabwe, Kenya, Nigeria, Argentina, Italy, Australia, Mexico, Japan, Poland, and New Zealand. Because none of the experiments reported the post-emergence application of fungicides, we assumed that hybrid differences in N uptake during grain filling were not confounded by non-treatment related attempts to prolong leaf stay green.

### Data analysis

The synthesized data set was divided into two eras, “Old” and “New,” with the Old Era containing all years prior to, and including, 1990 and the New Era covering 1991 to present. Because the primary objective of this study was to explore the change over time in how maize genotypes accumulate and utilize N, best efforts were made to assign data points to the Old or New Era based on when the genotypes used in the study were released and not necessarily when the research was conducted. If the genotype(s) used in the study was not described, or it was not possible to find the release date of the named genotype(s), the year the study was conducted was used to assign the data set to the appropriate era. Using year of genotype release to divide data into two eras leads to the possibility that the same experiment may contribute data to both eras (when multiple genotypes were used) and that some data points designated as Old Era were managed under New Era conditions. Approximately 17% of the data points in the Old Era arise from experiments conducted after 1991. The decision to divide the eras at the year 1990 was made in order that this review could be readily compared with the findings of other recent synthesis reviews (Ciampitti and Vyn, 2012, 2013); however it is important to note that those data sets were divided based on the year the research was conducted, not necessarily when the genotypes were released.

Whole plant above ground N uptake at R1 (R1N) and R6 (R6N) was the primary criteria for inclusion in this data set, but many other physiological parameters were also of interest and were included whenever possible. These parameters include continuous variables: Grain yield (GY), grain N content (GrainN), grain N concentration (GrainNc), and whole plant dry matter at R1 (R1DM) and R6 (R6DM), and plant density (PD). Categorical variables recorded were: irrigation, genotype, and N fertilizer source, application method, timing, and rate. Grain yields were adjusted to a 15.5% moisture content. If treatment means were reported as the average across several N rates, the average N rate was recorded.

Calculations for the N efficiency parameters of NUE, NIE, NRE, harvest index (HI), and N harvest index (NHI) were conducted using the equations presented in Ciampitti and Vyn (2012). Remobilized N (RemobN) was determined using the net delta equation *R1 N—(R6 N-Grain N)* (Ciampitti and Vyn, 2013). Post N accumulation was defined as the difference in N accumulation between R6 and R1.

Using the entire data set, select continuous variables (GY_P_, R6N_P_, GY_A_, R6N_A_, and NNI) were divided into groups to create categorical (Cat) variables. This division into similar groups allowed for more thorough evaluation of the response between the eras at different levels of GY and R6N. Likewise, the continuous variable NNI was used to divide the data set into separate categories of N stress at R1 to allow for comparison across eras. Separate assessment based on both g plant^−1^ and kg ha^−1^ was important to accurately explore the impact that PD had on these parameters. A similar division of the data set into categorical variables was used by Ciampitti and Vyn (2012). In this study GY_P_, R6N_P_, GY_A_, and R6N_A_ were each separated into four levels (1–4) divided at the lower quartile (25% Q), median, and upper quartile (75% Q) of each variable for the entire data set (Old and New Era combined). Cat-1 includes values less than the 25% Q, Cat-2 between 25% Q and the median, Cat-3 between median and 75% Q, and Cat-4 >75% Q. Similarly, the NNI was divided into N stress levels of Low, Med, and High by assigning values below 25% Q to HighStress, above 75% Q to LowStress, and values within the interquartile range (IQR) to MedStress. These categorical variables, as well as the N rates represented in each category, are defined in Table 1.

**Table 1:** **Criteria used to divide the continuous variables grain yield g plant^**−1**^ (GY_**P**_), whole plant N at R6 g plant^**−1**^ (R6N_**P**_), grain yield kg ha^**−1**^ (GY_**A**_), whole plant N at R6 kg ha^**−1**^ (R6N_**A**_), and NNI into categorical variables**.

**Categorical variable**	**Continuous variable**	**Criteria**	***N*** **Rates Included**	
			**Range**	**Mean**
			**— — –kg ha**^−1^**— — –**	
CatGY_A_-1	GY_A_	<7184	0–492	104
CatGY_A_-2	GY_A_	7184–9540	0–492	142
CatGY_A_-3	GY_A_	9540.1–11800	0–630	202
CatGY_A_-4	GY_A_	>11800	0–834	246
CatR6N_A_-1	R6N_A_	<136	0–492	79
CatR6N_A_-2	R6N_A_	136–179	0–450	148
CatR6N_A_-3	R6N_A_	179.1–225	0–450	194
CatR6N_A_-4	R6N_A_	>225	0–834	263
LowStress	NNI	>1.1	0–834	311
MedStress	NNI	1.1–0.8	0–450	182
HighStress	NNI	<0.8	0–492	106
			**— — –g plant**^−1^**— — –**	
CatGY_P_-1	GY_P_	<124	0–8.2	1.5
CatGY_P_-2	GY_P_	124–153	0–8.2	2.6
CatGY_P_-3	GY_P_	153.1–181	0–9.1	3.4
CatGY_P_-4	GY_P_	>181	0–13.9	4.8
CatR6N_P_-1	R6N_P_	<2.2	0–8.2	1.2
CatR6N_P_-2	R6N_P_	2.2–2.8	0–6.6	2.3
CatR6N_P_-3	R6N_P_	2.81–3.67	0–9.1	3.3
CatR6N_P_-4	R6N_P_	>3.67	0–13.9	4.8

*GY_P_, R6N_P_, GY_A_, and R6N_A_ categories (Cat) were divided into four groups (1–4) using the quartiles and the mean of each variable across both the Old and New Era. NNI was divided into three stress categories (Low, Med, High) using the interquartile range. The range and mean of N rates represented in each category are also reported*.

All statistical analyses were conducted using SAS 9.4 statistical software (SAS Institute, Cary NC, USA). Summary statistics were generated using the PROC MEANS procedure. Linear regressions were analyzed using PROC REG. Means separation for the categorical variables were conducted with use of a *t*-test in PROC GLM because of the uneven sample number within each variable. Sample sizes were uneven because not all variables recorded were reported in all papers used to compile this data set.

## Results and discussion

### Overview

Of the 711 data points collected from the 86 field experiments (Supplementary Tables 1, 2), 281 were assigned to the Old Era and 430 were assigned to the New Era. The mean N rate applied in the Old and New Era was 170 and 172 kg ha^−1^, respectively, and median N rates were also similar (Tables 2, 3). Because of the increase in average plant population (5.0 plants m^−2^ in the Old Era compared to 7.3 plants m^−2^ in the New Era) the mean N application rate per plant declined significantly from 3.6 to 2.4 g N plant^−1^ from the Old Era to the New Era (Tables 4, 5). N application rates were reported in all but six of the studies used (93%). It was more common in the New Era for the experimental design to include 0N control treatments (no N fertilizer applied). Of the 80 studies that reported N rates, the inclusion of 0N treatments was reported in only 14 experiments in the Old Era and in 25 experiments in the New Era.

**Table 2:** **Summary statistics based on per unit area for the Old Era data set**.

**Variable**	***N***	**Mean**	**Std Dev**	**Minimum**	**25% Q**	**Median**	**75% Q**	**Maximum**
PD_A_	281	50,316	18,506	22,660	33,991	53,000	65,745	111,940
GrainNc	214	1.50	0.30	0.61	1.30	1.51	1.72	2.16
%PostN	281	29.7	13.3	–2.7	22.1	28.9	38.3	70.4
%PostDM	157	51.1	12.6	–16.0	42.7	51.6	59.0	75.7
NIE	259	48.1	9.9	28.2	40.6	47.1	54.0	79.2
NHI	216	64.6	9.6	22.4	58.4	66.4	71.0	87.7
HI	174	50.8	8.9	18.3	44.7	49.9	56.6	76.5
R1NCE	156	67.9	22.1	39.0	52.9	60.2	72.9	138.1
R6NCE	156	96.8	21.4	44.6	82.5	90.4	111.1	155.1
%GrainNPostN	224	45.1	18.5	0.0	33.8	44.5	56.9	100.0
%R1Remob	216	49.4	13.6	7.6	41.8	50.0	59.4	87.0
StoverNc	133	0.66	0.27	0.20	0.49	0.64	0.77	2.41
NNI	161	0.96	0.24	0.33	0.83	1.00	1.13	1.34
%Post_StvDM_	139	13.4	19.8	–38.0	0.2	12.7	25.4	60.6
NRate_A_	275	170.3	102.1	0.0	105.0	170.0	225.0	492.0
R1N_A_	281	113.8	41.6	21.0	86.4	112.0	137.3	235.0
R6N_A_	281	165.1	62.3	35.6	123.7	157.6	203.6	386.8
GY_A_	263	7853.2	2826.5	1206.5	5642.5	7696.9	9715.2	19300.0
GrainN_A_	224	104.0	41.2	10.3	74.5	101.3	127.6	266.0
R1DM_A_	161	7324.0	2273.9	2059.2	6110.0	6866.2	8225.4	15372.0
R6DM_A_	192	15966.1	5165.0	4257.8	12168.3	15263.7	19529.6	31820.0
PostN_A_	281	51.4	35.2	0.0	25.5	44.0	67.0	188.3
PostDM_A_	157	8246.9	4076.6	–679.7	5071.6	7949.5	10000.0	21732.0
RemobN_A_	216	57.9	28.7	3.5	38.0	54.1	73.5	163.5
StoverN_A_	224	56.1	24.3	14.3	38.3	54.3	68.4	163.3
StoverDM_A_	182	9124.4	3183.8	2462.5	6854.8	8499.2	10774.0	19478.7
ΔStover_A_	139	1333.0	2612.8	–6467.1	–18.6	833.7	2079.6	11380.7

*Units in kg ha^−^^1^ are denoted with the subscript “_A._” PD, Plant density; GrainNc, grain N concentration; %PostN, proportion of total N accumulated after silking; %PostDM, proportion of dry matter accumulated after silking; NIE, N internal efficiency; NHI, N harvest index; HI, harvest index; %GrainNPostN, proportion of grain N arising from post-silking N accumulation; %R1Remob, proportion of R1 N remobilized to the grain; StoverNc, stover N concentration; NNI, N Nutrition Index; %Post_StvDM_, percent change in stover dry weight from silking to maturity; Nrate, N rate applied; R1N, N accumulated at silking; R6N, N accumulated at physiological maturity; GY, grain yield at 15.5% moisture; GrainN, grain N content; R1DM, whole-plant dry matter accumulated at silking; R6DM, whole-plant dry matter accumulated at physiological maturity; PostN, N accumulated after silking; PostDM, dry matter accumulated after silking; RemobN, N remobilized to the grain; StoverN, stover N content; StoverDM, stover dry matter at physiological maturity; ΔStover, change in stover dry matter from silking to physiological maturity*.

**Table 3:** **Summary statistics based on per unit area for the New Era data set**.

**Variable**	***N***	**Mean**	**Std Dev**	**Minimum**	**25% Q**	**Median**	**75% Q**	**Maximum**
PD_A_	419	73,396	14,664	50,000	60,000	75,000	82,500	104,000
GrainNc	271	1.25	0.33	0.32	1.08	1.28	1.42	2.27
%PostN	427	36.4	11.9	5.5	28.5	36.3	44.3	66.5
%PostDM	281	52.3	9.0	30.5	45.4	53.4	59.0	73.2
NIE	332	55.8	13.4	32.6	47.7	54.2	60.9	129.5
NHI	290	61.4	9.2	29.8	54.9	62.0	67.7	84.3
HI	248	56.2	9.2	35.0	49.5	55.9	60.9	88.8
R1NCE	281	77.8	20.8	32.7	64.4	72.8	89.7	140
R6NCE	281	102.4	19.2	56.8	88.2	99.6	115.3	167.5
%GrainNPostN	291	57.0	19.0	7.3	44.0	57.5	70.1	100.0
%R1Remob	287	27.4	13.2	0.5	17.8	26.8	36.3	70.7
StoverNc	199	0.77	0.32	0.28	0.60	0.71	0.88	2.95
NNI	281	0.92	0.25	0.42	0.74	0.90	1.07	1.70
%Post_StvDM_	233	6.5	20.6	–84.3	–3.1	7.3	19.3	55.9
NRate_A_	427	171.8	140.8	0.0	67.0	165.0	240.0	834.0
R1N_A_	427	123.7	46.8	36.9	87.6	117.1	154.0	303.0
R6N_A_	427	195.0	63.5	47.0	147.0	192.6	235.0	407.6
GY_A_	335	10564.2	2628.9	3754.1	8454.8	10648.0	12395.0	18988.7
GrainN_A_	292	124.1	44.6	28.2	89.2	119.7	154.0	287.7
R1DM_A_	281	9295.5	2219.9	4001.0	7500.0	9314.0	10754.2	15794.8
R6DM_A_	290	19566.4	3994.3	9900.0	16770.0	19861.5	22060.0	33475.5
PostN_A_	427	71.3	33.6	5.0	46.9	67.0	91.0	202.1
PostDM_A_	281	10378.1	2973.5	3300.0	8150.0	10196.0	12300.0	18292.5
RemobN_A_	287	54.6	31.4	1.0	31.4	49.2	73.0	186.9
StoverN_A_	292	78.0	31.5	17.0	55.3	73.0	95.4	194.0
StoverDM_A_	252	10183.1	3103.9	2947.4	8478.4	10110.9	11538.8	22034.1
ΔStover_A_	239	793.8	2156.8	–5157.0	–455.9	578.2	1974.8	7491.3

*Units in kg ha ^−^^1^ are denoted with the subscript “_A._” PD, Plant density; GrainNc, grain N concentration; %PostN, proportion of total N accumulated after silking; %PostDM, proportion of dry matter accumulated after silking; NIE, N internal efficiency; NHI, N harvest index; HI, harvest index; %GrainNPostN, proportion of grain N arising from post-silking N accumulation; %R1Remob, proportion of R1 N remobilized to the grain; StoverNc, stover N concentration; NNI, N Nutrition Index; %Post_StvDM_, percent change in stover dry weight from silking to maturity; Nrate, N rate applied; R1N, N accumulated at silking; R6N, N accumulated at physiological maturity; GY, grain yield at 15.5% moisture; GrainN, grain N content; R1DM, whole-plant dry matter accumulated at silking; R6DM, whole-plant dry matter accumulated at physiological maturity; PostN, N accumulated after silking; PostDM, dry matter accumulated after silking; RemobN, N remobilized to the grain; StoverN, stover N content; StoverDM, stover dry matter at physiological maturity; ΔStover, change in stover dry matter from silking to physiological maturity*.

**Table 4:** **Summary statistics based on per plant determinations for the Old Era data set**.

**Variable**	***N***	**Mean**	**Std Dev**	**Minimum**	**25% Q**	**Median**	**75% Q**	**Maximum**
NRate_P_	275	3.6	2.4	0.0	2.0	3.3	4.7	9.4
R1N_P_	281	2.5	1.1	0.3	1.7	2.3	3.1	7.6
R6N_P_	281	3.6	1.6	0.5	2.5	3.4	4.4	10.0
GY_P_	263	172.7	72.6	25.6	125.8	166.4	217.0	432.8
GrainN_P_	224	2.32	1.10	0.19	1.45	2.05	3.07	5.76
R1DM_P_	161	151.2	49.2	46.8	108.4	144.7	187.0	316.0
R6DM_P_	192	317.4	91.5	116.4	258.9	311.4	368.6	607.0
PostN_P_	281	1.11	0.79	0.00	0.55	0.89	1.54	4.95
PostDM_P_	157	166.2	67.5	–27.2	121.0	156.0	210.0	372.0
RemobN_P_	215	1.3	0.6	0.1	0.8	1.2	1.7	3.0
StoverN_P_	224	1.28	0.76	0.26	0.77	1.08	1.72	4.90
StoverDM_P_	182	181.8	53.4	56.0	137.6	184.0	219.0	318.8
ΔStover_P_	139	27.8	38.9	–42.9	0.3	20.0	48.2	160.1

*Units in g plant^−1^ are denoted with the subscript “_P._” Nrate, N rate applied; R1N, N accumulated at silking; R6N, N accumulated at physiological maturity; GY, grain yield at 15.5% moisture; GrainN, grain N; R1DM, dry matter accumulated at silking; R6DM, whole-plant dry matter accumulated at physiological maturity; PostN, N accumulated after silking; PostDM, dry matter accumulated after silking; RemobN, N remobilized to the grain; StoverN, stover N content; StoverDM, stover dry matter at physiological maturity; ΔStover, change in stover dry matter from silking to physiological maturity*.

**Table 5:** **Summary statistics on a per plant basis for the New Era data set**.

**Variable**	***N***	**Mean**	**Std Dev**	**Minimum**	**25% Q**	**Median**	**75% Q**	**Maximum**
NRate_P_	419	2.4	2.0	0.0	1.0	2.2	3.6	13.9
R1N_P_	422	1.7	0.6	0.5	1.2	1.6	2.0	4.0
R6N_P_	422	2.7	0.9	0.9	2.1	2.6	3.2	6.1
GY_P_	326	146.7	36.2	54.8	121.9	148.6	170.5	253.2
GrainN_P_	286	1.71	0.63	0.51	1.24	1.63	2.04	3.84
R1DM_P_	276	124.4	29.3	59.3	104.7	120.7	134.9	263.2
R6DM_P_	282	264.1	56.4	116.5	225.0	260.1	300.4	447.2
PostN_P_	422	1.0	0.5	0.1	0.6	0.9	1.3	2.7
PostDM_P_	271	141.31	43.09	38.82	109.20	139.63	173.23	247.17
RemobN_P_	281	0.8	0.4	0.0	0.4	0.7	1.0	2.5
StoverN_P_	286	1.09	0.47	0.26	0.76	1.01	1.37	3.08
StoverDM_P_	246	139.5	43.7	58.3	114.5	131.2	160.5	367.2
ΔStover_P_	233	12.3	27.1	–58.9	–4.2	9.4	28.9	97.9

*Units in g plant ^−^^1^ are denoted with the subscript “_P._” Nrate, N rate applied; R1N, N accumulated at silking; R6N, N accumulated at physiological maturity; GY, grain yield at 15.5% moisture; GrainN, grain N content; R1DM, whole-plant dry matter accumulated at silking; R6DM, whole-plant dry matter accumulated at physiological maturity; PostN, N accumulated after silking; PostDM, dry matter accumulated after silking; RemobN, N remobilized to the grain; StoverN, stover N content; StoverDM, stover dry matter at physiological maturity; ΔStover, change in stover dry matter from silking to physiological maturity*.

When evaluating the distribution of the data set as a whole, the size of IQR was contingent on whether the comparison being made was in g plant^−1^ or kg ha^−1^. Most notably, the IQR was much wider for the Old Era than the New Era for the parameters of GY_P_ and R6N_P_ (Tables 4, 5), but the IQR for GY_A_ and R6N_A_ was quite similar between eras (Tables 2, 3). This may be partially explained by the sorting of “old” hybrids grown under “new” plant densities into the Old Era, but more likely it is an insight into the impact of higher plant populations on per plant performance and the move toward genotypes that are less variable in their response across environments and stress levels (Tollenaar and Wu, 1999). Because it is very difficult to separate out the population effects, changes over time in terms of g plant^−1^ (_P_)and kg ha^−1^ (_A_) will be discussed separately. Due to the linear conversion between kg ha^−1^ and g plant^−1^, when a value is changed to a proportion (ex. %PostN = PostN_A_/R6N_A_), the proportion is the same regardless of the units used for the calculation (kg ha^−1^ or g plant^−1^). Therefore, where appropriate, proportions will be used in order to avoid the population bias.

### Changing plant N dynamics

The New Era accumulated significantly more R6N_A_ than the Old Era (195 kg ha^−1^, *n* = 427 and 165 kg ha^−1^, *n* = 281, respectively; Tables 2, 3). However, more important than the change in total N accumulation is the change in when N is taken up and how this impacts whole-plant N dynamics throughout the growing season. Overall, the New Era took up a significantly greater proportion of its N after silking compared to the Old Era (36.4%, *n* = 427 and 29.7%, *n* = 281, respectively). Likewise, in the New Era the proportion of GrainN that arose from PostN increased to 57.0% (*n* = 291) from 45.1% (*n* = 224) in the Old Era (Tables 2, 3). The Old Era mean of 45.1% of GrainN originating from PostN agrees with other reports from that era of PostN contributing 40–50% of the N in the grain at maturity (Hay et al., 1953; Crawford et al., 1982; Osaki et al., 1991). More recently, Ciampitti and Vyn (2013) reported PostN contributed 50 and 56% of the GrainN in the Old and New Eras, respectively (eras were also divided at year 1991). This leads to an important observation that a smaller percentage of R1N is remobilized to the grain (%R1Remob) in the New Era.

To evaluate the impact of GY and R6N level on %R1Remob in more detail, the categorical variables of CatR6N_P_ and CatGY_P_ were used. These comparisons were made at the plant scale because the number of data points from each Era were more evenly distributed within categories compared to those based on a per unit area basis (CatR6N_A_and CatGY_A_). For both CatR6N_P_ and CatGY_P_, there was no consistent pattern within Eras across the groups. However, the Old Era was significantly higher than the New Era within all levels except for the highest CatGY_P_(GY_P_ > 3.67 g plant^−1^), where there was no difference between eras. This illustrates that when categorized by total N accumulation or final GY, the New Era is capable of achieving the same quantity (R6N or GY) with a smaller %R1Remob. The decline of %R1Remob in the New Era was offset by an increase in %PostN, similar to findings by Coque and Gallais (2007) when evaluating NUE across 23 European maize hybrids.

From the Old to New Era, the overall NHI declined significantly from 64.6% (*n* = 216) to 61.4% (*n* = 290) which contrasts with the significant increase in HI from 50.8% in the Old Era (Table 2) to 56.2% in the New Era (Table 3). The previous findings of Ciampitti and Vyn (2012) found no change in NHI between eras and a smaller increase in HI (47.6–49.8%). The NHI decline was at least partially due to the significant loss in GrainNc (1.5%, *n* = 214 to 1.2%, *n* = 271 for the Old and New Eras, respectively), although it should be noted that experimental results in wheat have shown NHI to not be directly related to GrainNc (Sinclair, 1998; Gastal et al., 2015). The decline of GrainNc in modern hybrids has been documented in earlier studies (Chen et al., 2013; Ciampitti and Vyn, 2013). Because the increase in GY_A_ has been greater than the decline in GrainNc, the total GrainN_A_ was still significantly higher in the New Era (124 kg ha^−1^, *n* = 292) compared to the Old Era (104 kg ha^−1^, *n* = 224). Several authors have reported an inherent inverse relationship between grain yield and GrainNc (Simmonds, 1995; Scott et al., 2006; Uribelarrea et al., 2007; Gallais et al., 2008). This could be attributed to the greater energy requirement for protein synthesis compared to starch synthesis (Penning de Vries et al., 1974).

A second contributing factor to the decline over time in the NHI was that modern genotypes maintained a higher StoverNc through physiological maturity (0.75%, *n* = 199 in the New Era relative to 0.66%, *n* = 133 in the Old Era). Increased stay-green, related to delayed N remobilization and leaf senescence (Ta and Weiland, 1992; Rajcan and Tollenaar, 1999a; Ciampitti and Vyn, 2011), causes more N to remain in the stover (and not be transported to the grain) at the end of the growing season. Although the stay-green trait has played an important role in increasing grain yield in maize, it is also important to acknowledge that leaf senescence is not an entirely negative process. Because some of the N fixed in the senescing leaf will be remobilized to the developing grain (He et al., 2004; Chen et al., 2015a), leaves that remain green but do not maintain active photosynthesis (Thomas and Howarth, 2000; Hörtensteiner, 2009) provide no yield benefit.

### Drivers of PostN uptake

Previous literature suggests that the increase in total plant N uptake over time has largely been driven by the increase in total biomass accumulation (Below, 2002; Hirel et al., 2007). This hypothesis is supported by this data set because of strong correlations between total R6DM_A_ and R6N_A_ accumulation for the Old Era (*R*^2^ = 0.77, *n* = 192) and the New Era (and *R*^2^ = 0.47, *n* = 290), albeit with the Old Era having significantly steeper slope (relationship not shown). Likewise, the PostN_A_ also appeared to have been driven by PostDM_A_ in the Old Era (*R*^2^ = 0.64, *n* = 157), but this relationship is considerably weaker in the New Era (*R*^2^ = 0.22, *n* = 281). These findings imply that N and DM accumulation during grain fill are not as tightly linked in the New Era compared to the Old Era. Over two decades ago, Moll et al. (1994) found a positive linear relationship between PostN_P_ and PostDM_P_ when comparing three hybrids across three N rates. PostN_A_ was weakly correlated to GY_A_ in this data set in both eras (*R*^2^ = 0.30, *n* = 595 for the Old and New Era combined), but comparatively more of the variation in PostN_A_ was explained by GrainN_A_ (*R*^2^ = 0.52, *n* = 516 Old and New Eras combined).

Interestingly, two factors that did not drive PostN_A_ were R1N_A_ (*R*^2^ = 0.08, *n* = 708 for the Old and New Era combined) and RemobN_A_ (*R*^2^ = 0.004, *n* = 503 for Old and New Era combined). This differs from results analyzed by Coque and Gallais (2007) who found R1N_A_ to be predictive of PostN_A_ under adequate N fertilization in an experiment utilizing 23 commercial and experimental hybrids. Additionally, Ciampitti and Vyn (2013) reported a positive linear relationship between RemobN_A_ and R1N_A_ (*R*^2^ = 0.60, *n* = 503 for Old and New Era combined), indicating that a larger pool of N already in the plant at the onset of reproductive stages is related to a larger amount of N being remobilized from vegetative tissue to the ear during grain fill. Although we found no relationship between the quantitative values of PostN_A_ and RemobN_A_, previous research overwhelmingly reports an antagonistic relationship between these two sources of GrainN (Pan et al., 1984, 1995; Rajcan and Tollenaar, 1999a; Mi et al., 2003; He et al., 2004; Pommel et al., 2006; Gallais et al., 2007; Ciampitti and Vyn, 2011; Ciampitti et al., 2013). In light of this large body of evidence suggesting that PostN and RemobN have an inverse relationship, it is possible that we did not realize such a relationship because of our greater inclusion of more recent hybrids and the larger variation in genotypes and environments represented in this data set (Supplementary Table 1).

Another factor impacting PostN that was not directly accounted for in this data set is the activity of the root system as the corn progresses through the reproductive stages. Previous literature has hypothesized that PostN is limited by the amount of photo assimilates transported to the roots (Pan et al., 1995; Rajcan and Tollenaar, 1999a), in agreement with the interdependence of root N uptake on carbohydrate supply from the shoot (Raper et al., 1978). While studying prolific hybrids, Pan et al. (1995) suggested that the developing ear and root systems compete for photo assimilates and, therefore, PostN is inversely related to ear strength. To quantify this, researchers calculated the net change in stover dry weight during the grain filling period (ΔStover = [PostDM-GrainDM]) and found a positive, linear relationship between these values and PostN_P_. This is to say, the greater the amount of PostDM allocated to the grain (i.e., when ΔStover is small), the higher the sink strength of the ear, and the less PostN accumulated. This relationship appears to hold true in this data set, but only in the Old Era. While there was a positive relationship between PostN_P_ and ΔStover_P_ in the Old Era (*R*^2^ = 0.41, *n* = 139), in the New Era ΔStover_P_ explains none of the variation in PostN_P_ (*R*^2^ = 0.05, *n* = 233). These results suggest that PostN does not have an inverse relationship with sink strength in modern genotypes. One explanation for this shift is the well-documented persistence of the stay-green trait in modern genotypes (Ma and Dwyer, 1998; Valentinuz and Tollenaar, 2004; Echarte et al., 2008). If photosynthesis is maintained longer into the reproductive stages, this may prevent such strong competition for nutrients and carbohydrates between the roots and the developing ear, allowing for simultaneously high ear strength with high PostN accumulation.

### NIE

Previously it was stated that the average N rate was not significantly different between the Old and New Era (170 and 172 kg ha^−1^, respectively), while the average plant density increased from 5.0 to 7.3 plants m^−2^. This dictates that while plant populations have been increasing, the quantity of N applied per plant has declined. It has also been established that New Era genotypes generally out-yield the Old Era genotypes on a per area basis. The latter implies that modern genotypes are able to produce a greater GY_A_ with lower N inputs. In this data set, there were too few points for which we were able to calculate NUE for accurate interpretation because of the need for a 0N control treatment, but we were able to investigate NIE. The mean NIE [*GY (15.5% moisture)/R6N*] of the New Era (55.8, *n* = 332) is significantly higher than the mean NIE of the Old Era (48.1, *n* = 259). These respective NIE values for the two eras are similar to values previously reported by Ciampitti and Vyn (2012). Under the different levels of CatR6N_A_, GY_A_ increased significantly between each level within an Era, and within each level the New Era is significantly higher than the Old Era (relationship not shown). The New Era is clearly superior in producing GY_A_ at each given level of R6N_A_; hence the large increase in NIE.

On a per plant basis, the difference in GY_P_ between Eras at each level of CatR6N_P_ was less drastic. Again, both eras significantly increased GY_P_ between each CatR6N_P_ level. However, in this scenario the only significant differences between eras at a given CatR6N_P_ occurred at the lowest and highest levels (relationship not shown). At the lowest level (CatR6N_P_-1), the New Era mean GY_P_ was significantly higher than the Old Era. This relationship was reversed at the highest level (CatR6N_P_-4) where the Old Era significantly out-yielded the New Era. This discrepancy may be explained, in part, by the impact of the range of plant densities represented within these categories. The range of plant density was greater in the Old Era compared to the New Era (IQR = 31,754 and IQR = 22,500, respectively), partially due to the inclusion of data points from modern experiments utilizing hybrids released before 1991 (and therefore assigned to the Old Era). As mentioned previously, this creates the “problem” of having some Old Era genotypes grown under New Era management (fertilizer rates, plant population, etc.). The latter (and especially the plant population influence) also helps to explain how the New Era appears to have a lower maximum, and also a higher minimum, GY_P_ (Table 5). However, it is beyond the scope of this review to attempt to separate out those influences.

As discussed by Gastal et al. (2015), NIE at R6 can be further broken down into two components: the ability of the plant to produce biomass per unit of N accumulated (N conversion efficiency, R6NCE), and the ability of the plant to convert accumulated dry matter to harvestable grain (harvest index, HI). The explanation presented by Gastal et al. (2015) suggests that R6NCE is highly conserved among species of the same metabolic group when compared at the same biomass, and therefore, most of the variation in NIE should be explained by HI rather than NCE. However, within this complex data set (gathered across a very wide range of decades, environments, genotypes and management treatments with varying original sample sizes for biomass estimation) the more dominant influence of HI vs. NCE on NIE did not hold true. Mean NCE was significantly higher for the New Era genotypes at both the R1 and R6 growth stages (Tables 2, 3). Furthermore, when considering the data set as a whole, HI only explained 24% (*R*^2^ = 0.24, *n* = 418) of the variation seen in NIE (relationship not shown). In contrast, R6NCE explained 51% of the variation in NIE (*R*^2^ = 0.51, *n* = 373) when both Eras were combined (relationship not shown). For both HI and R6NCE there was no significant difference between the slopes of the two Eras when regressed against NIE. Furthermore, because R6NCE was not correlated to R6DM_A_ (*R*^2^ = 0.03, *n* = 437 for both the Old and New Era combined) it does not appear that the improved R6NCE in modern hybrids is solely the result of an increase in total biomass production seen in the New Era. This more detailed analysis further supports the above interpretation that much of the noted increase in NIE is the result of an increased efficiency in modern hybrids to accumulate biomass per unit of N uptake.

### Response to N stress

One of the most intriguing questions about hybrid era influences is that of the direction and mechanisms of any changes over time in N stress tolerance during the growing season. This data set allowed us to evaluate physiological responses to varying levels of N stress by quantifying the whole-plant NNI at R1 (Sadras and Lemaire, 2014). These NNI values were then separated into three levels: LowStress (NNI > 1.1), MedStress (1.1 > NNI > 0.8), and HighStress (NNI < 0.8). Sadras and Lemaire (2014) explain that crop N status can be considered non-limiting when NNI > 1 (i.e., the biomass yield would not increase further with increased N supply). Likewise, an NNI < 1 signifies crop biomass status is limited by N supply. Under this definition, the MedStress category encompasses the transition from N limited to non-N limited.

**Figure 1:**
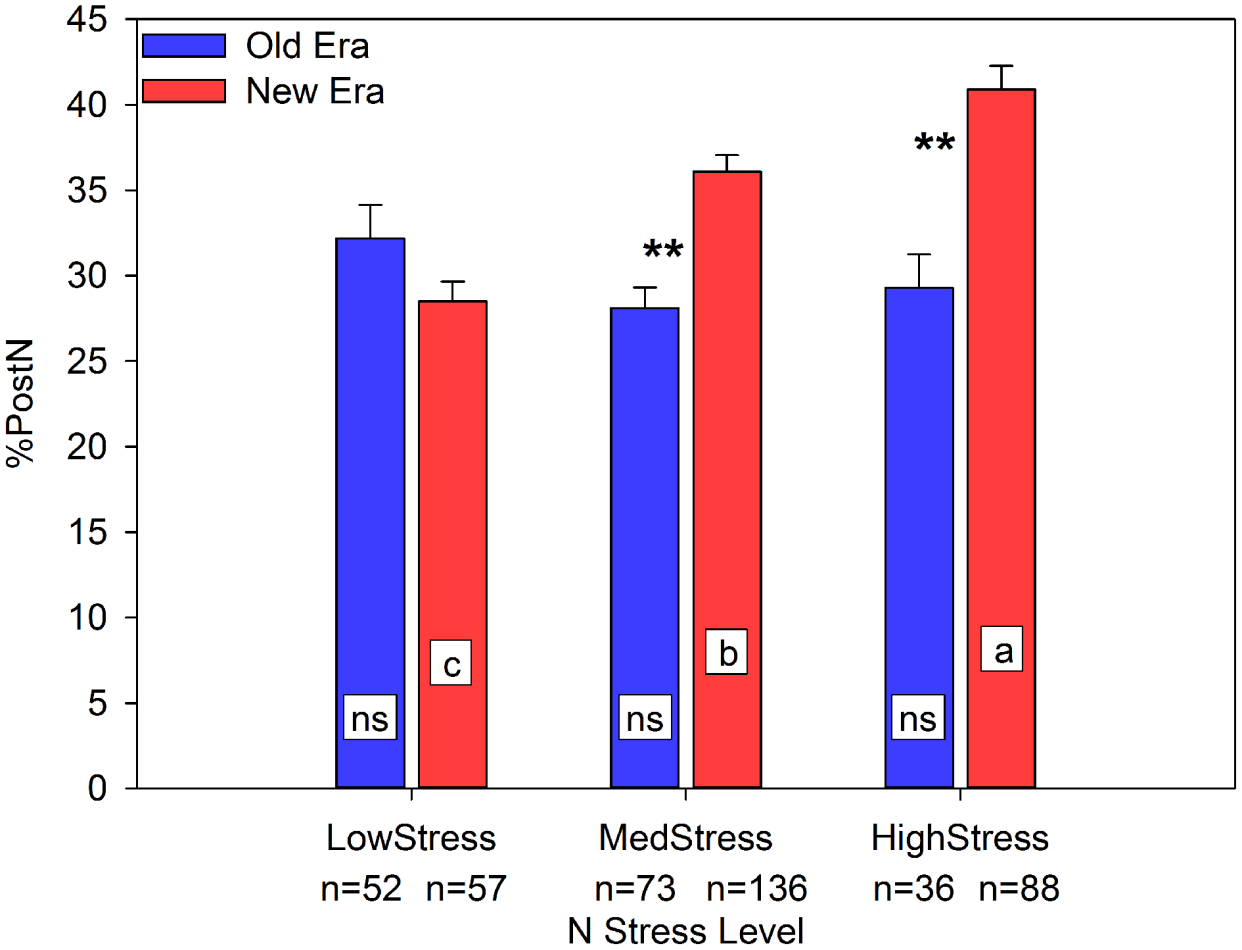
**Relationship between N stress level, as determined by NNI at R1, and percent of total N accumulated post-silking (%PostN) across maize genotypes in the Old and New Eras**. Different letters signify LSMeans within Eras (Old or New) are significantly different across N stress levels. An ^**^denotes a significant difference between the Old and New Era LSMeans within a given N stress level. ^**^denotes significance at the 0.01 probability level.

NNI was a useful predictor of R6N_A_ in both the Old and New Eras (*R*^2^ = 0.65, *n* = 161 and *R*^2^ = 0.55, *n* = 281, respectively), with a significant difference between the regression slopes of the two Eras. NNI also had a positive linear relationship with RemobN_A_ (*R*^2^ = 0.50, *n* = 290, Old and New Era combined). This relationship illustrates that as the R1 N status of the maize crop increases, the amount of N remobilized (in kg ha^−1^) also increases, although there was no significant difference between the two eras. As discussed previously, this data set suggests that over time the primary source of GrainN has shifted from RemobN to PostN. In support of that concept, Figure 1 clearly shows that one mechanism New Era genotypes employ to maintain yield under N stress conditions is an increase in the %PostN. Under LowStress, there was no Era difference in the %PostN. However, at both MedStress and HighStress, the New Era accumulated a significantly greater proportion of its total N after silking than the Old Era. Further, there was a steady increase in %PostN for the New Era across the three N stress levels, while the Old Era had no significant change among N stress levels. Similarly, the proportion of GrainN arising from PostN followed much the same pattern in response to N stress as did the %PostN (relationship not shown). This finding agrees with those of Ta and Weiland (1992) and De Oliveira Silva (2015) who found using enriched ^15^N that greater proportions of any ^15^N uptake by plants were allocated to the kernels as the growing season progressed. If a greater proportion of the GrainN under N stress conditions originates from concurrent N uptake, this indicates a pattern of delayed N remobilization and increased photosynthetic duration through the reproductive period.

**Figure 2:**
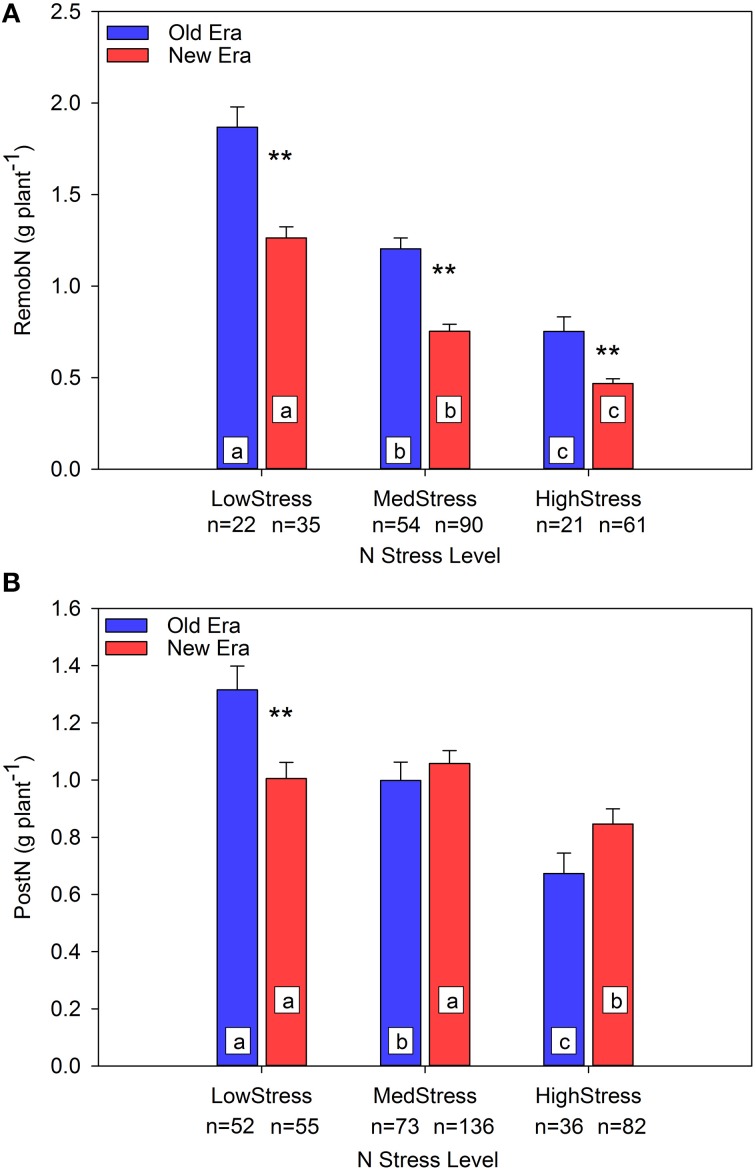
**Relationship between N stress level, as determined by NNI at R1, and remobilized N (RemobN) (A) and post-silking N uptake (PostN) (B) in g plant^**−1**^ for the Old and New Eras**. Different letters signify LSMeans within Eras (Old or New) are significantly different across N stress levels. An ^**^denotes a significant difference between the Old and New Era LSMeans within a given N stress level. ^**^denotes significance at the 0.01 probability level.

To further evaluate the mechanisms underlying the observed pattern in %PostN response to N stress, an evaluation in absolute terms of the impact of N stress on both PostN and RemobN is also valuable. Prior studies have documented that, under N stress, maize maintains leaf area at the sacrifice of N uptake per unit leaf area (Vos et al., 2005; Lemaire et al., 2008b). This strategy maintains resource capture at the expense of resource use efficiency, and also reduces the pool of N accumulated during the vegetative stages that is available to be remobilized to the developing grain during reproductive growth. Unfortunately, leaf area was seldom documented in the studies included in this data set. Figure 2A illustrates that RemobN_P_ declines as N stress increases in both Eras, and that at each level of N stress the New Era remobilized significantly less N than the Old Era. In the Old Era, PostN_P_ also declines significantly across each level of N stress, but the New Era only shows a significant decrease in PostN_P_ at the HighStress level (Figure 2B). Thus, although the absolute value of RemobN_P_ declines with increasing N stress for both Eras, PostN_P_ uptake is better maintained under N stress in the New Era compared to the Old Era. On a per area basis, RemobN_A_ decreased significantly for both Eras across all three levels of N stress, and at each level there were no significant differences between the Eras (relationship not shown). PostN_A_ followed a similar pattern as PostN_P_ with the exception that the New Era is significantly higher than the Old Era at the MedStress level (relationship not shown).

**Figure 3:**
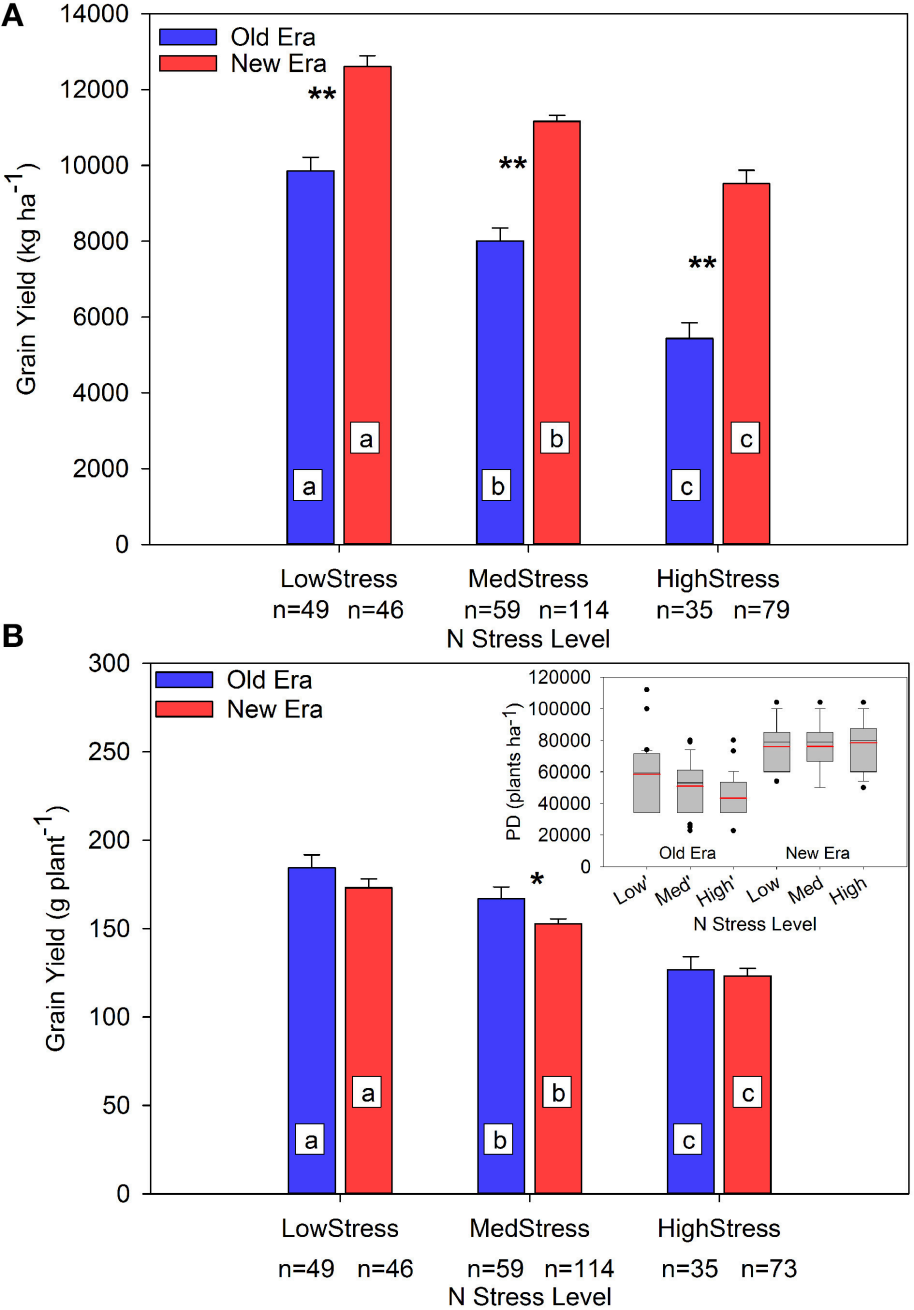
**Relationship between N stress level and grain yield (kg ha^**−1**^) (A) and grain yield (g plant^**−1**^) (B) across maize genotypes in the Old and New Eras as determined by R1 NNI**. Different letters signify LSMeans within Eras (Old or New) are significantly different across N stress levels. An ^*^denotes a significant difference between the Old and New Era LSMeans within a given N stress level. ^*^denotes significance at the 0.05 probability level. ^**^denotes significance at the 0.01 probability level. The sub-figure in panel **(B)** represents the distribution of plant density (PD) within each group. Black lines represent the group median PD and red lines represent the group mean PD.

The impact of the increase in N stress level on GY is presented in Figure 3. The GY_A_ decreased significantly in both eras as N stress level increased, and the era GY_A_'s were significantly different from each other within each level. The interpretation of this information is complicated by the analysis of GY_P_ where once again the main effect of N stress level on GY_P_ was significant at each level, but the only significant interaction between eras occurred under MedStress where the Old Era GY_P_ was significantly higher than that of the New Era.

The sub-plot in Figure 3B shows the range of PDs present in each N stress category. The New Era PDs were consistent across all N stress levels. On the contrary, average PD in the Old Era decreased significantly as N stress increased. The significantly higher average PD and wide IQR at LowStress vs. HighStress in the Old Era was not caused by the inclusion of data originating from studies conducted post-1991 but using Old Era genotypes since only 1 data point from the LowStress level originated from a study conducted post-1991. Instead, the most likely explanation is the presence of high-yield studies in the Old Era that utilized above normal plant populations and N rates in those decades. That said, it is noteworthy that the New Era achieved the same GY_P_ as the Old Era under LowStress and HighStress despite experiencing much higher population stress (New Era mean PD was from 23 to 45% higher than the Old Era across the three N stress levels). This is evidence of an increase in stress tolerance across both N availability and PD levels, which can be, at least partially, attributed to the New Era's increased ability to accumulate N post-silking. The increased tolerance to crowding stress and increased yield stability has also been noted by others (Tollenaar and Wu, 1999; Tollenaar and Lee, 2002), but this is the first report of more modern genotypes having such substantially higher plant N stress tolerance even in the middle of the growing season.

**Figure 4:**
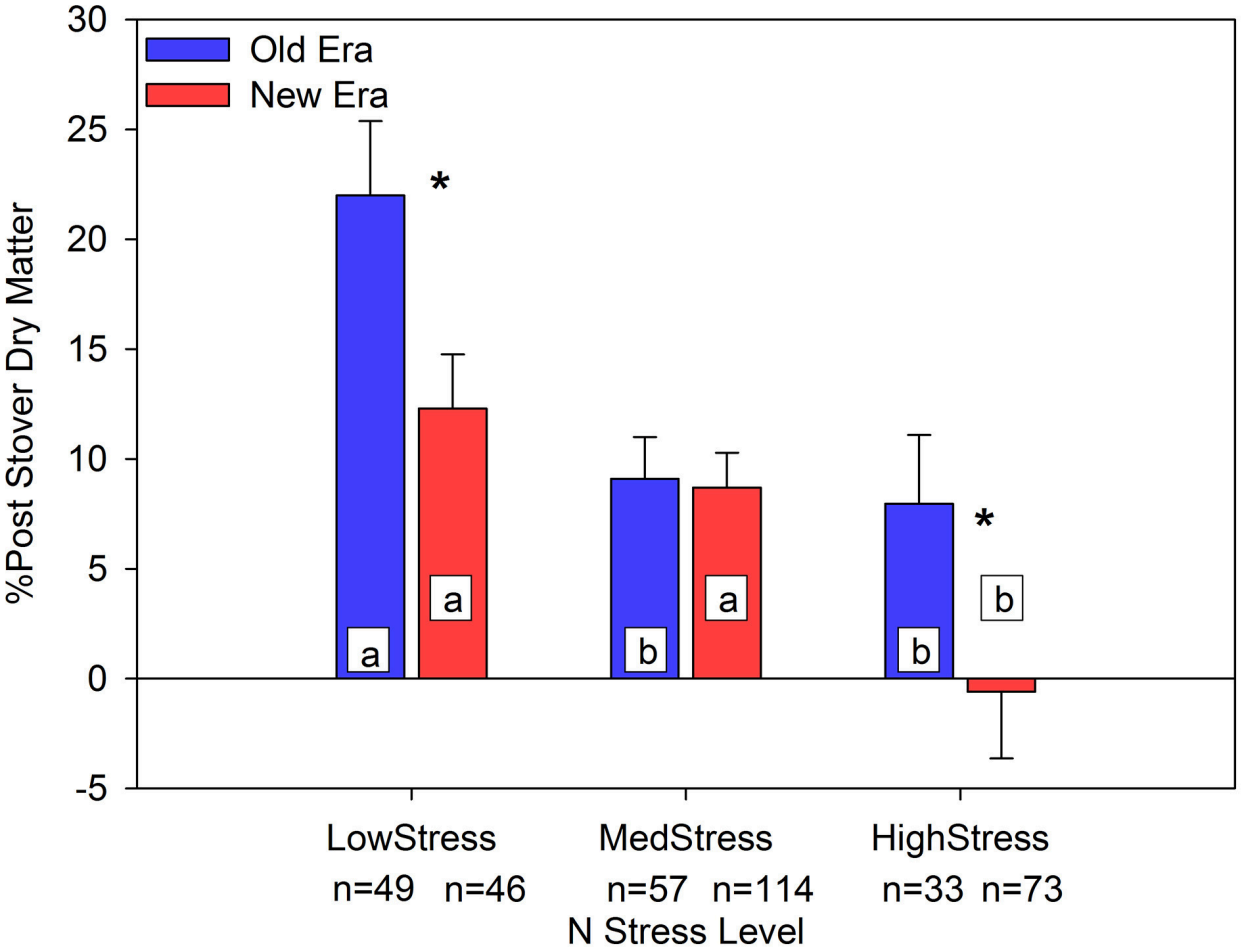
**Relationship between N stress level, as determined by NNI at R1, and the percent change in stover dry matter from R1 to R6 (%PostStover DM) across maize genotypes in the Old and New Eras**. Different letters signify LSMeans within Eras (Old or New) are significantly different across N stress levels. An ^*^denotes a significant difference between the Old and New Era LSMeans within a given N stress level. ^*^denotes significance at the 0.05 probability level.

Another important change in response to N stress was the proportion of PostDM that was partitioned to the grain compared to the stover. Building on the concept of ΔStover discussed by Pan et al. (1995), we calculated the percent change in StoverDM from R1 to R6 (%Post_StvDM_) as *(PostDM-GrainDM)/R6StoverDM*. The overall means of %Post_StvDM_ in the Old and New Eras were 13.4% (*n* = 139) and 6.5% (*n* = 233), respectively, with the Old Era being significantly higher. Although both Eras declined in %Post_StvDM_ as N stress increased, the New Era was only significantly lower than the Old Era at the LowStress and the HighStress levels (Figure 4). This suggests that although the New Era resulted in an increase in StoverNc as well as a greater proportion of N left in the stover at physiological maturity (1-NHI), the amount of PostDM partitioned to the stover (instead of to the grain) was less in the New Era than in the Old Era, especially under N stress.

**Figure 5:**
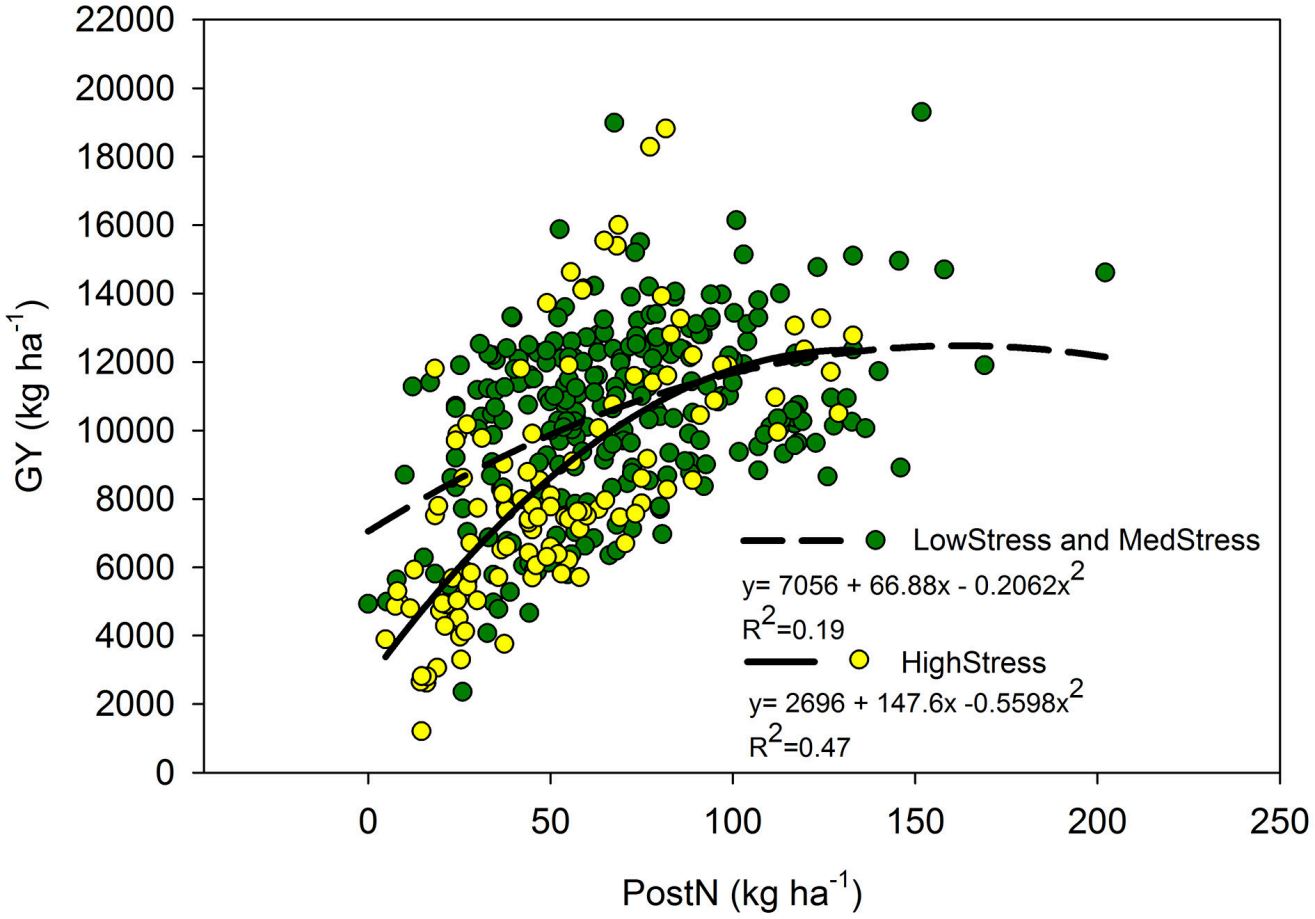
**Quadratic relationship between post-silking N (kg ha^**−1**^) and grain yield (kg ha^**−1**^) by N stress level as determined by NNI at the R1 stage**. The LowStress and MedStress lines were combined because there was no significant difference between these lines.

Our utilization of N stress levels also proved useful in the interpretation of the role PostN plays in determining final GY. Although PostN_A_was not predictive of GY_A_ overall, the relationships were more clear when evaluated by N stress level. Under HighStress (but not Med or LowStress), PostN_A_ was correlated with GY_A_ (Figure 5), whereas at LowStress R1N was more predictive of GY_A_ than Med or HighStress (relationship not shown). It is unclear as to whether this is indicative of the plasticity of the maize crop or a further indication of the inverse relationship between PostN and sink size. These relationships agree with findings of Akintoye et al. (1999) who found PostN to be a better indicator of final GY than R1N under high N stress conditions across six genotypes. There were too few observations available in the separate eras for the era impacts of N stress on PostN, R1N, and GY to be clarified further.

## Summary and conclusions

There has been much speculation as to the changing role of PostN in maize over time, and how this may impact the way modern genotypes are managed agronomically. This synthesis analysis provides strong evidence that as N stress level at the R1 stage increases, hybrids in the New Era increased in %PostN while hybrids within the Old Era did not change (Figure 1). At the same time, the New Era also maintained a similar GY_P_ within both the Low and High stress categories, despite being grown at significantly higher plant populations (Figure 3B). This is evidence of modern genotypes exhibiting greater resilience to both N and PD stresses. Modern hybrids also appeared to be better able to convert more of their resources to grain development and less to stover DM, as shown in Figure 4. A smaller gain in StoverDM during the reproductive stages may suggest more efficient allocation of carbohydrates assimilated during reproductive growth to the grain.

The relationship between GY_A_ and PostN_A_ appeared to be dependent on N stress level (Figure 5). When the data set was considered as a whole, PostN_A_ was a poor predictor of GY_A_, but the correlation was much stronger under the HighStress N stress level compared to the Med and LowStress levels. Likewise, R1N_A_ was more strongly correlated with GY_A_ under LowStress as compared to Med and HighStress. It was not clear from this analysis whether this was further evidence of the enhanced resiliency of modern hybrids to excel in their environments, or further evidence of the inverse relationship between sink strength and PostN (because sink strength is smaller under high N stress).

PostN uptake appeared to be driven by PostDM gains in the Old Era (*R*^2^ = 0.64, *n* = 157) but less so in the New Era (*R*^2^ = 0.22, *n* = 281). Although numerous previous studies, including published papers utilized in this data set, have reported an inverse relationship between PostN and RemobN, we did not find these GrainN sources to be antagonistic to one another. This may be due to the inclusion of more recent hybrids in the New Era and the larger variation in genotypes and environments included in this data set. Using ΔStover as a means of estimating sink strength, we found a strong correlation between ΔStover_P_ and PostN_P_ in the Old Era (*R*^2^ = 0.41, *n* = 139), but not in the New Era (*R*^2^ = 0.05, *n* = 233). This suggests that photosynthetic assimilation of modern hybrids is better able to meet ear demands while simultaneously maintaining plant function during grain fill.

This review further supports previous findings that modern hybrids take up a significantly greater proportion of their total N after silking (36.4%, *n* = 427) compared to hybrids released prior to 1991 (29.7%, *n* = 281; Tables 2, 3). The New Era hybrids relied on a larger proportion of GrainN originating from PostN compared to RemobN (Tables 2, 3) even as GY_A_ increases. NIE also increased from 48.1 (*n* = 259) in the Old Era to 55.8 (*n* = 332) in the New Era because of the considerable combined gains in both NCE and HI, but especially because of the increase in NCE. New Era genotypes possess superior ability to produce higher total biomass and GY_A_ even when similar levels of R6N_A_ are accumulated. One crop metric that had declined over time was NHI (64.6, *n* = 216–61.4, *n* = 290 from the Old to New Era, respectively). This NHI decline occurred despite the increase in HI in the New Era, and was the result of a simultaneous decrease in GrainNc and increase in StoverNc (likely due to persistence of leaf area longer into the reproductive stages).

Clearly, modern genotypes take up a greater proportion of their N after silking, and this has positively impacted their ability to produce higher yields. This review provides persuasive evidence that this later-season N uptake advantage is most evident under N stressed conditions. Further research is needed to explore how this physiological trait can be measured in large-scale breeding programs and how it can be exploited by maize farmers to more efficiently manage N nutrition.

## Author contributions

SM Data and reference collection, synthesis data analysis, stress category assignment for all maize plant dry matter and nitrogen data, construction of tables, figures, and appendix summary of data sources, co-author and joint corresponding author. TV Co-author, guidance on data selection, references, data summarization and analysis, selections of major plant nutrition and plant physiology parameter relationships to be explored, manuscript theme clarifications, selection of sub-themes, and primary corresponding author.

### Conflict of interest statement

The authors declare that the research was conducted in the absence of any commercial or financial relationships that could be construed as a potential conflict of interest.
