# Supplementary material for: Maize Plant Resilience to N Stress and Post-silking N Capacity Changes over Time: A Review
Source: Front Plant Sci. 2016 Feb 9;7:53. doi: 10.3389/fpls.2016.00053 (PMC4746326; doi:10.3389/fpls.2016.00053)
Supplement: Supplementary file 1 [file Table1.pdf]

Supplementary Table 1
Published references used in compilation of synthesized data set. Listed by primary author, year(s) study was conducted, N rate(s) applied (kg ha^{-1}), plant density (PD) (plant m^{-2}), and country where research was conducted.

No.	Author	Year Study was Conducted	N rate (kg ha^{-1})	PD (plant m^{-2})	Country
1	Akintoye et al.	1991, 1992	0, 70, 140, 210	5.3	Nigeria
2	Anderson et al.	Ex. A: 1980, 1981	56, 112, 168, 224	2.3, 3.4	USA
		Ex. B: 1979, 1980	56, 224	2.3	USA
3	Antonietta et al.	2008, 2009, 2010	100, 120	6.0, 7.0, 8.0, 9.0, 10.0	Argentina
4	Barber et al.	1987, 1988, 1989	336	7.3, 7.1	USA
5	Beauchamp et al.	1971	145	4.4	Canada
6	Bender et al.	2010	202	8.4	USA
7	Bigeriego et al.	1976	0	7.0	USA
8	Boyer	2007, 2008	168	4.0, 5.4, 6.4, 7.4, 8.4	USA
9	Bundy et al.	1985, 1986	0, 78, 157, 235,	2.5, 2.7	USA
10	Chen et al.	2012, 2013	55, 220	5.4, 7.9, 10.4	USA
11	Chen et al.	2010, 2011, 2012, 2013	60, 120, 180, 240	6.0	China
12	Chen et al.	2010, 2011	240	6.0	China
13	Chen et al.	2010, 2011	0, 120, 240	6.0	China
14	Cimapitti et al.	2010, 2011	0, 112, 224	5.4, 7.9, 10.4	USA
15	Ciampitti et al.	2009	0, 165, 330	5.4, 7.9, 10.4	USA
16	Cirilo et al.	2003, 2004	0, 250	8.0	Argentina
17	Coque et al.	2002, 2003	90, 200	10.0	France
18	Cui et al.	2010, 2011	300	6.8	China
19	Cui et al.	2011	225, 450	8.8	China
20	De Oliveira Silva	2012, 2013	0, 112	8.0	USA
21	Di Fonzo et al.	1978, 1979	100, 350	5.9	Italy
22	Eghball et al.	1989	0, 60, 120, 180	5.3	USA
23	Gentinetta et al.	1982	300	6.0	Italy
24	Goodroad et al.	1983, 1984	30, 110, 190, 350	6.6	USA
25	Grzebisz et al.	2001, 2002, 2003	135	Not Reported	Poland
26	Hanway	1959	0, 44, 88, 176	4.3	USA
27	Hay et al.	1945	0	4.3	USA
28	He et al.	2000	0, 250, 400	6.0	China

29	Hou et al.	Ex. A: 2007, 2008<br>Ex. B: 2009	0, 209, 225, 450<br>225	6.6, 8.9, 9.0<br>6.5	China<br>China
30	Jin et al.	2010, 2011	0, 126, 160, 185, 225, 300, 450	6.0, 7.5, 8.7	China
31	Jing et al.	2009, 2010	0, 360, 405, 450, 495, 540	8.3	China
32	Johnson et al.	1962, 1964	124	6.3	USA
33	Jones et al.	1903	Not reported	2.5	USA
34	Kamprath et al.	Not reported	56, 168, 280	3.7	USA
35	Karlen et al.	1980, 1982	250, 268	7.0, 10.0	USA
36	Karlen et al.	1985	560	11.2	USA
37	Kosgey et al.	2008, 2009	0, 50, 150, 200, 270	10.0	New Zealand
38	Kovács	2010, 2011, 2012	0, 90, 145, 202	8.5	USA
39	Lafitte et al.	1989, 1990, 1991	0, 200	5.3	Mexico
40	Lemcoff et al.	Not reported	0, 167	3.6, 7.3	USA
41	Li et al.	2007, 2008	Not reported	5.2, 7.5	China
42	Liu et al.	2009, 2010, 2011	0, 100, 200, 250, 300, 400	8.5	China
43	Lü et al.	2008, 2009	0, 100	7.5	China
44	Ma et al.	1993, 1994, 1995	0, 42, 59, 100	5.8	Canada
45	Martin et al.	2002	170	9.9	France
46	Mi et al.	1996, 1997	45, 225	6.0	China
47	Miller	2010, 2011	0, 45, 90, 135, 180, 225	8.2	USA
48	Moll et al.	1978	56, 224	2.5	USA
49	Moll et al.	1990, 1991	56, 140, 224	3.4	USA
50	Montemurro et al.	2000, 2001, 2002	0, 100, 200	6.7	Italy
51	Muchow	1986	0, 60, 120, 240, 420	7.0	Australia
52	Ning et al.	2009, 2010	200	6.0	China
53	Oikeh et al.	1993, 1994	0, 30, 90, 120	5.3	Nigeria
54	Osaki	1982, 1984	0, 100, 150, 300	5.3, 11.1	Mexico
55	Osaki et al.	1988	100	5.6	Japan
56	Pan et al.	1981, 1982	56, 224	3.4	USA
57	Pan et al.	1983, 1984	275, 308, 411	3.4, 4.5	USA
58	Peng et al.	2005	150	6.0	China
59	Peng et al.	2007, 2008, 2009	0, 230, 250, 395, 450	10.0	China
60	Pommel et al.	2001, 2002	30, 170	Not Reported	France
61	Qi et al.	2010, 2011	466	7.5	China

62	Rajcan et al.	1993, 1994, 1995	0, 150	7.3	Canada
63	Reed et al.	1978	200	4.6	USA
64	Ren et al.	2011, 2012	300	6.8	China
65	Sayre	1940	Not reported	2.7	USA
66	Subedi et al.	2003, 2004	0, 150	7.5	Canada
67	Swank et al.	1981	0, 12	5.9	USA
68	Ta et al.	1989	67, 257	6.0	USA
69	Tsai et al.	1986, 1987	268	5.3	USA
70	Tsai et al.	1979, 1980	0, 201, 447	5.4	USA
71	Tsai et al.	1986, 1987	67, 134, 268	5.5	USA
72	Uribelarrea et al.	2001, 2002, 2003	0, 34, 68, 102, 170, 204, 238	6.5	USA
73	Wang et al.	2010	492	6.0	China
74	Wang et al.	2007	0, 300	7.5	China
75	Wang et al.	2010, 2011	200, 247, 300, 474	6.0, 7.5, 8.3	China
76	Wang et al.	2011, 2012	0, 120, 240	5.0	China
77	Wolfe et al.	1983	0, 180	7.0	USA
78	Worku et al.	2003, 2004	0, 18, 90, 168	5.3	Zimbabwe, Kenya
79	Xiao et al.	Not reported	225	8.4	China
80	Yan et al.	2010, 2011	0, 30, 45, 56, 92, 250	7.1, 7.5, 7.6	China
81	Yibrin et al.	1992, 1993	0, 340	7.7	USA
82	Zhan et al.	2009	0, 195, 240, 280	Not Reported	China
83	Zhang et al.	2010, 2011	0, 225	6.7	China
84	Zhao et al.	2005, 2006	0, 357, 417, 626, 360, 714, 834	6.0	China

Supplementary Table 2

Full citations of references used in compilation of synthesized data set.

No.	Citation
1	Akintoye, H.A., E.O. Lucas, and J.G. Kling. 1999. Grain yield and yield components of single, double, and synthetic maize lines grown at four N levels in three ecological zones of West Africa. Trop. Agric. 76(1): 51–56.
2	Anderson, E.L., E.J. Kamprath, and R.H. Moll. 1985. Prolificacy and N fertilizer effects on yield and N utilization in maize. Crop Sci. 25(4): 598.
3	Antonietta, M., D.D. Fanello, H.A. Acciaresi, and J.J. Guiamet. 2014. Senescence and yield responses to plant density in stay green and earlier-senescing maize hybrids from Argentina. F. Crop. Res. 155: 111-119.
4	Barber, K.L., L.D. Maddux, G.M. Pierzynski, D.E. Kissel, and B.R. Bock. 1992. Corn responses to ammonium- and nitrate-nitrogen fertilization. Soil Sci. Soc. Am. J. 56(4): 1166.
5	Beauchamp, E.G., L.W. Kannenberg, and R.B. Hunter. 1976. Nitrogen accumulation and translocation in corn genotypes following silking. Agron. J. 68(2): 418.
6	Bender, R.R., J.W. Haegele, M.L. Ruffo, and F.E. Below. 2013. Nutrient uptake, partitioning, and remobilization in modern, transgenic insect-protected maize hybrids. Agron. J. 105: 161–170.
7	Bigeriego, M., R.D. Hauck, and R. a. Olson. 1979. Uptake, Translocation and Utilization of 15N-Depleted Fertilizer in Irrigated Corn. Soil Sci. Soc. Am. J. 43(3): 528.
8	Boyer, M.J. 2013. Dry matter and nutrient accumulation in corn hybrids from the 1960's to 2000's. M.S. Thesis. Iowa State University.
9	Bundy, L.G., and P.R. Carter. 1988. Corn hybrid response to nitrogen fertilization in the Northern Corn Belt. J. Prod. Agric. 1(2): 99.
10	Chen, K., S. V. Kumudini, M. Tollenaar, and T.J. Vyn. 2015a. Plant biomass and nitrogen partitioning changes between silking and maturity in newer versus older maize hybrids. F. Crop. Res. 183: 315–328.
11	Chen, Y., C. Xiao, D. Wu, T. Xia, Q. Chen, F. Chen, L. Yuan, and G. Mi. 2015b. Effects of nitrogen application rate on grain yield and grain nitrogen concentration in two maize hybrids with contrasting nitrogen remobilization efficiency. Eur. J. Agron. 62: 79–89.
12	Chen, Y., C. Xiao, X. Chen, Q. Li, J. Zhang, F. Chen, L. Yuan, and G. Mi. 2014. Characterization of the plant traits contributed to high grain yield and high grain nitrogen concentration in maize. F. Crop. Res. 159: 1–9.
13	Chen, X., F. Chen, Y. Chen, Q. Gao, X. Yang, L. Yuan, F. Zhang, and G. Mi. 2013. Modern maize hybrids in Northeast China exhibit increased yield potential and resource use efficiency despite adverse climate change. Glob. Chang. Biol. 19(3): 923–936.
14	Ciampitti, I.A., S.T. Murrell, J.J. Camberato, M. Tuinstra, Y. Xia, P. Friedemann, and

T.J. Vyn. 2013. Physiological dynamics of maize nitrogen uptake and partitioning in response to plant density and nitrogen stress factors: II. Reproductive phase. Crop Sci. 53: 2588–2602.

15 Ciampitti, I.A., and T.J. Vyn. 2011. A comprehensive study of plant density consequences on nitrogen uptake dynamics of maize plants from vegetative to reproductive stages. F. Crop. Res. 121: 2–18.

16 Cirilo, A.G., J. Dardanelli, M. Balzarini, F.H. Andrade, M. Cantarero, S. Luque, and H.M. Pedrol. 2009. Morpho-physiological traits associated with maize crop adaptations to environments differing in nitrogen availability. F. Crop. Res. 113(2): 116–124.

17 Coque, M., and A. Gallais. 2007. Genetic variation among European maize varieties for nitrogen use efficiency under low and high nitrogen fertilization. Maydica 52: 383–397.

18 Cui, C., J. Gao, X. Yu, Z. Wang, J. Sun, S. Hu, Z. Su, and M. Xie. 2013a. Dry matter accumualtion and nitrogen migration of high-yielding spring maize for different nitrgoen efficiency in the flowering and milking stages. J. Plant Nutr. Fertil. 19(6): 1337–1345.

19 Cui, H., L. Jin, B. Li, S. Dong, P. Liu, B. Zhao, and J.-W. Zhang. 2013b. Effects of shading on dry matter accumulation and nutrient absorption of summer maize. Chinese J. Appl. Ecol. 24(11): 3099–3105.

20 De Oliveira Silva, A. 2015. Evaluation of nitrogen use effeiciency (NUE) in maize genotypes using in-season soil applications of isotopic nitrogen in field plots. M.S. Thesis. Purdue University.

21 Di Fonzo, N., M. Motto, T. Maggiore, R. Sabatino, and F. Salamini. 1982. N-uptake, translocation and relationships among N-related traits in maize as affected by genotype. Agronomie 2(9): 789–796.

22 Eghball, B., and J.W. Maranville. 1991. Interactive effects of water and nitrogen stresses on nitrogen utilization efficiency, leaf water status and yield of corn genotypes. Commun. Soil Sci. Plant Anal. 22(13-14): 1367–1382.

23 Gentinetta, E., D. Ceppl, C. Lepori, G. Perico, M. Motto, and F. Salamini. 1986. A major gene for delayed senescence in maize. Pattern of photosynthates accumulation and inheritance. Plant Breed. 97(3): 193–203.

24 Goodroad, L.L., and M.D. Jellum. 1988. Effect of N fertilizer rate and soil pH on N efficiency in corn. Plant Soil 106: 85–89.

25 Grzebisz, W., M. Wronska, J.B. Diatta, and P. Dullin. 2008. Effect of zinc foliar application at an early stage of maize growth on patterns of nutrients and dry matter accumulation by the canopy. Part II. Nitrogen uptake and dry matter accumulation patterns. J. Elem. 13(1): 29–39.

26 Hanway, J.J. 1962a. Corn growth and composition in relation to soil fertility: I. Growth of different plant parts and relaton between leaf weight and grain yield. Agron. J.

54: 145–148.

Hanway, J.J. 1962b. Corn growth and composition in relation to soil fertility: II. Uptake of N, P, and K and their distribution in different plant parts during the growing season. Agron. J. 54: 217–222.

27 Hay, R.E., E.B. Earley, and E.E. Deturk. 1953. Concentration and translocation of nitrogen compounds in the corn plant (Zea mays) during grain development. Plant Physiol. 28(4): 606–621.

28 He, P., W. Zhou, and J. Jin. 2004. Carbon and nitrogen metabolism related to grain formation in two different senescent types of maize. J. Plant Nutr. 27(2): 295–311.

29 Hou, P., Q. Gao, R. Xie, S. Li, Q. Meng, E. a. Kirkby, V. Römheld, T. Müller, F. Zhang, Z. Cui, and X. Chen. 2012. Grain yields in relation to N requirement: Optimizing nitrogen management for spring maize grown in China. F. Crop. Res. 129: 1–6.

30 Jin, L.B., H.Y. Cui, B. Li, J.S. Yang, S.T. Dong, B. Zhao, P. Liu, and J.W. Zhang. 2013. Effects of integrated agronomic practices on nitrogen efficiency and soil nitrate nitrogen of summer maize. Acta Agron. Sin. 39(11): 2009–2015.

31 Jing, L.Q., F.C. Zhao, D.C. Wang, J.H. Yuan, D.L. Lu, and W.P. Lu. 2013. Effects of nitrogen application on accumulation and distribution of nitrogen, phosphorus, and potassium of summer maize under super-high yield conditions. Acta Agron. Sin. 39(8): 1478–1490.

32 Johnson, R.R., K.E. McClure, L.J. Johnson, E.W. Klosterman, and G.B. Triplett. 1966. Corn plant maturity. I. Changes in dry matter and protein distribution in corn plants. Agron. J. 58(2): 151–153.

33 Jones, W.J., J., and H.A. Huston. 1914. Composition of maize at various stages of its growth. Indiana Agric. Exp. Stn. Bull. 175.

34 Kamprath, E.J., R.H. Moll, and N. Rodriguez. 1982. Effects of nitrogen fertilization and recurrent selection on performance of hybrid populations of corn. Agron. J. 74: 955–958.

35 Karlen, D.L., E.J. Sadler, and C.R. Camp. 1987. Dry matter, nitrogen, phosphorus, and potassium accumulation rates by corn on norfolk loamy sand. Agron. J. 79: 649–656.

36 Karlen, D.L., R.L. Flannery, and E.J. Sadler. 1988. Aerial accumulation and partitioning of nutrients by corn. Agron. J. 80(2): 232.

37 Kosgey, J.R., D.J. Moot, a. L. Fletcher, and B. a. McKenzie. 2013. Dry matter accumulation and post-silking N economy of “stay-green” maize (Zea mays L.) hybrids. Eur. J. Agron. 51: 43–52.

38 Kovács, P. 2013. Systems evaluation of shallow anhydrous ammonia placements, rates, and timing on maize plant uniformity, yield, and N use efficiency. Ph.D. Dissertation. Purdue University.

39 Lafitte, H.R., and G.O. Edmeades. 1994a. Improvement for tolerance to low soil

nitrogen in tropical maize II. Grain yield, biomass production, and N accumulation. F. Crop. Res. 39: 15–25.

Lafitte, H.R., and G.O. Edmeades. 1994b. Improvement for tolerance to low soil nitrogen in tropical maize III. Variation in yield across environments. F. Crop. Res. 39(1): 27–38.

40 Lemcoff, J.H., and R.S. Loomis. 1994. Nitrogen and density influences on silk emergence, endosperm development, and grain yield in maize (Zea mays L.). F. Crop. Res. 38(2): 63–72.

41 Li, C.F., M. Zhao, P. Liu, J.W. Zhang, J.S. Yang, and S. Dong. 2014. Characteristics of grain filling and nitrogen translocation of maize parent lines released in different eras in China. Acta Agron. Sin. 40(11): 1990–1998.

42 Liu, J., A. Zhan, L. Bu, L. Zhu, S. Luo, X. Chen, Z. Cui, S. Li, R.L. Hill, and Y. Zhao. 2014. Understanding dry matter and nitrogen accumulation for high-yielding film-mulched maize. Agron. J. 106(2): 390–396.

43 Lü, P., J. Zhang, W. Liu, J. Yang, P. Liu, S. Dong, and D. Li. 2011. Effects of nitrogen application on yield and nitrogen use efficiency of summer maize in super-high yield conditions. J. Plant Nutr. Fertil. Sci. 17(5): 1099–1107.

44 Ma, B.L., and L.M. Dwyer. 1998. Nitrogen uptake and use of two contrasting maize hybrids differing in leaf senescence. Plant Soil 199: 283–291.

45 Martin, A., X. Belastegui-Macadam, I. Quilleré, M. Floriot, M.H. Valadier, B. Pommel, B. Andrieu, I. Donnison, and B. Hirel. 2005. Nitrogen management and senescence in two maize hybrids differing in the persistence of leaf greenness: Agronomic, physiological and molecular aspects. New Phytol. 167(2): 483–492.

46 Mi, G., J. Liu, F. Chen, F. Zhang, Z. Cui, and X. Liu. 2003. Nitrogen uptake and remobilization in maize hybrids differing in leaf senescence. J. Plant Nutr. 26(1): 237–247.

47 Miller, E. 2012. Nitrogen application timing and rate effects on nitrogen utilization of corn and the adoption of active optical refelectance sensors for nitrogen management. M.S. Thesis. Purdue University.

48 Moll, R.H., E.J. Kamprath, and W.A. Jackson. 1982. Analysis and interpretation of factors which contribute to efficiency of nitrogen utilization. Agron. J. 74(3): 562.

49 Moll, R.H., W.A. Jackson, and R.L. Mikkelsen. 1994. Recurrent selection for maize grain yield: Dry matter and nitrogen accumulation and partitioning changes. Crop Sci.: 874–881.

50 Montemurro, F., M. Maiorana, D. Ferri, and G. Convertini. 2006. Nitrogen indicators, uptake and utilization efficiency in a maize and barley rotation cropped at different levels and sources of N fertilization. F. Crop. Res. 99: 114–124.

51 Muchow, R.C. 1988a. Effect of nitogen supply on the comparative productivity of maize and sorghum in a semi-arid tropical environment III. Grain yield and nitrogen

accumulation. F. Crop. Res. 18: 31–43.

Muchow, R.C. 1988b. Effect of nitrogen supply on the comparative productivity of maize and sorghum in a semi-arid tropical environment I. Leaf growth and leaf nitrogen. F. Crop. Res. 18: 1–16.

52 Ning, P., S. Li, P. Yu, Y. Zhang, and C. Li. 2013. Post-silking accumulation and partitioning of dry matter, nitrogen, phosphorus and potassium in maize varieties differing in leaf longevity. F. Crop. Res. 144: 19–27.

53 Oikeh, S.O., R.J. Carsky, J.G. Kling, V.O. Chude, and W.J. Horst. 2003. Differential N uptake by maize cultivars and soil nitrate dynamics under N fertilization in West Africa. Agric. Ecosyst. Environ. 100: 181–191.

54 Osaki, M. 1995. Comparison of productivity between tropical and temperate maize I. Leaf senescence and productivity in relation to nitrogen nutrition. Soil Sci. Plant Nutr. 41(3): 439–450.

55 Osaki, M., T. Shinano, and T. Tadano. 1991. Redistribution of carbon and nitrogen compounds from the shoot to the harvesting organs during maturation in field crops. Soil Sci. Plant Nutr. 37(1): 117–128.

56 Pan, W.L., E.J. Kamprath, R.H. Moll, and W.A. Jackson. 1984. Prolificacy in corn: Its effects on nitrate and ammonium uptake and utilization. Soil Sci. Soc. Am. J. 48(5): 1101.

57 Pan, W.L., J.J. Camberato, R.H. Moll, E.J. Kamprath, and W.A. Jackson. 1995. Altering source-sink relationships in prolific maize hybrids: Consequences for nitogen uptake and remobilization. Crop Sci. 35(3): 836–845.

58 Peng, Y., J. Niu, Z. Peng, F. Zhang, and C. Li. 2010. Shoot growth potential drives N uptake in maize plants and correlates with root growth in the soil. F. Crop. Res. 115(1): 85–93.

59 Peng, Y., X. Li, and C. Li. 2012. Temporal and spatial profiling of root growth revealed novel response of maize roots under various nitrogen supplies in the field. PLoS One 7(5): e37726.

60 Pommel, B., A. Gallais, M. Coque, I. Quilleré, B. Hirel, J.L. Prioul, B. Andrieu, and M. Floriot. 2006. Carbon and nitrogen allocation and grain filling in three maize hybrids differing in leaf senescence. Eur. J. Agron. 24(3): 203–211.

61 Qi, W., X. Chen, P. Liu, G. Li, L. Shao, F. Wang, S. Dong, J. Zhang, and B. Zhao. 2013. Characteristics of dry matter, accumulation and distribution of N, P and K of super-high-yield summer maize. J. Plant Nutr. Fertil. Sci. 19(1): 26–36.

62 Rajcan, I., and M. Tollenaar. 1999a. Source : sink ratio and leaf senescence in maize: II. Nitrogen metabolism during grain filling. F. Crop. Res. 60: 255–265.

Rajcan, I., and M. Tollenaar. 1999b. Source : sink ratio and leaf senescence in maize: I. Dry matter accumulation and partitioning during grain filling. F. Crop. Res. 60: 255–265.

63 Reed, A. J., F.E. Below, and R.H. Hageman. 1980. Grain protein accumulation and the relationship between leaf nitrate reductase and protease activities during grain development in maize (Zea mays L.): I. Variation between genotypes. Plant Physiol. 66: 164–170.

64 Ren, B., J. Zhang, X. Li, X. Fan, S. Dong, P. Liu, and B. Zhao. 2014. Effect of watterlogging on nutrient uptake and transport of summer maize. J. Plant Nutr. Fertil. Sci. 20(2): 298–308.

65 Sayre, J.D. 1948. Mineral Accumulation in Corn. Plant Physiol. 23(3): 267–281.

66 Subedi, K.D., and B.L. Ma. 2007. Dry matter and nitrogen partitioning patterns in Bt and non-Bt near-isoline maize hybrids. Crop Sci. 47: 1186–1192.

67 Swank, J.C., F.E. Below, R.J. Lambert, and R.H. Hageman. 1982. Interaction of carbon and nitrogen metabolism in the productivity of maize. Plant Physiol. 70: 1185–1190.

68 Ta, C.T., and R.T. Weiland. 1992. Nitrogen partitioning in maize during ear development. Crop Sci. 32(2): 443.

69 Tsai, C.L., and C.Y. Tsai. 1990. Endosperm modified by cross-pollinating maize to induce changes in dry matter and nitrogen accumulation. Crop Sci. 30(4): 804–808.

70 Tsai, C.Y., D.M. Huber, D. V. Glover, and H.L. Warren. 1984. Relationship of N deposition to grain yield and N response of three maize hybrids. Crop Sci. 24: 277–281.

71 Tsai, C., D.M. Huber, H.L. Warren, and C.Y. Tsai. 1991. Effects of cross-pollination on dry matter accumulation , nutrient partitioning and grain yield of maize hybrids grown under different levels of N fertility. J. Sci. Food Agric. 57: 163–174.

72 Uribelarrea, M., S.P. Moose, and F.E. Below. 2007. Divergent selection for grain protein affects nitrogen use in maize hybrids. F. Crop. Res. 100(1): 82–90.

73 Wang, X., Y. Cao, W. Wei, L. Zhang, Y. Wang, S. Bian, and L. Wang. 2012. Characteristics of dry matter production and nitrogen use efficiency of 37 spring maize hybrids with high-yielding potential in north of China. J. Plant Nutr. Fertil. Sci. 18(1): 60–68.

74 Wang, Y.L., C.H. Li, J.F. Tan, X. Zhang, and T.X. Liu. 2011. Effect of postponing N application on yield, nitrogen absorption and utilization in super-high-yield summer maize. Acta Agron. Sin. 37(2): 339–347.

75 Wang, Z., J. Gao, and B.L. Ma. 2014. Concurrent improvement in maize yield and nitrogen use efficiency with integrated agronomic management strategies. Agron. J. 106: 1243–1250.

76 Wang, Z., J. Li, and Y. Li. 2014. Effects of drip system uniformity and nitrogen application rate on yield and nitrogen balance of spring maize in the North China Plain. F. Crop. Res. 159: 10–20.

77 Wolfe, D.W., D.W. Henderson, T.C. Hsiao, and A. Alvino. 1988. Interactive water and

nitrogen effects on senescence of maize. I. Leaf area duration, nitrogen distribution, and yield. Agron. J. 80: 859–864.

78 Worku, M., M. Bänziger, G.S.A. Erley, D. Friesen, A.O. Diallo, and W.J. Horst. 2007. Nitrogen uptake and utilization in contrasting nitrogen efficient tropical maize hybrids. Crop Sci. 47(2): 519–528.

79 Xiao, Q., L. Yan, X. Zhu, H. Zhang, B. Cao, X. Ni, L. Li, J. Yang, D. Huang, and W. Yi. 2014. Dynamic analysis of dry matter and NPK accumulation with time in summer maize. J. Plant Nutr. Fertil. Sci. 20(3): 606–612.

80 Yan, P., S. Yue, M. Qiu, X. Chen, Z. Cui, and F. Chen. 2014. Using maize hybrids and in-season nitrogen management to improve grain yield and grain nitrogen concentrations. F. Crop. Res. 166: 38–45.

81 Yibirin, H., J.W. Johnson, and D. Eckert. 1996. Corn production as affected by daily fertilization with ammonium, nitrate, and phosphorus. Soil Sci. Soc. Am. J. 60: 512–518.

82 Zhan, X., T. Li, X. Han, D. Zou, R. Zou, and B. Ye. 2011. Effects of nitrogen fertilization methods on yield, profit and nitrogen absorption and utilization of spring maize. J. Plant Nutr. Fertil. Sci. 17(4): 861–868.

83 Zhao, B., S.T. Dong, J.W. Zhang, and P. Liu. 2010. Effects of controlled-release fertilizer on yield and nitrogen accumulation and distribution in summer maize. Acta Agron. Sin. 36(10): 1760–1768.

84 Zhao, B., S.-T. Dong, J.-W. Zhang, and P. Liu. 2010. Effects of Controlled-Release Fertilizer on Yield and Nitrogen Accumulation and Distribution in Summer Maize. Acta Agron. Sin. 36(10): 1760–1768.
